# Detection of Paroxysmal Atrial Fibrillation in Stroke/Tia Patients

**DOI:** 10.1155/2013/840265

**Published:** 2013-03-26

**Authors:** Muhib Khan, Daniel J. Miller

**Affiliations:** ^1^Department of Neurology, University of Massachusetts, USA; ^2^Department of Neurology, Henry Ford Hospital, USA

## Abstract

One-third of stroke and transient ischemic attack (TIA) are cryptogenic, and paroxysmal atrial fibrillation (PAF) has been suggested as a possible cause for these cryptogenic strokes. Multiple studies have recently evaluated long-term cardiac rhythm monitoring with good yield for PAF. The duration of monitoring varies between studies as well as the qualifying event definition. Moreover, the clinical significance of very brief atrial fibrillation events is unclear in the literature. This paper provides an overview of current advances in the detection of paroxysmal atrial fibrillation, the clinical and genetic factors predictive of arrhythmia detection, and the therapeutic dilemma concerning this approach.

## 1. Introduction 

One-third of stroke and transient ischemic attack (TIA) are cryptogenic requiring additional investigation and intervention [1]. Occult paroxysmal atrial fibrillation (PAF) has been suggested as a possible cause for these cryptogenic strokes [2]. Atrial fibrillation has been long associated with high risk of stroke, but most of this knowledge is derived from patient data from chronic atrial fibrillation. It has been suggested that PAF is more prevalent than persistent atrial fibrillation in stroke and TIA patients [3]. Anticoagulation therapy initiated after detection of atrial fibrillation (AF) provides an additional 40% risk reduction of stroke as compared to antiplatelet therapy alone [4].

Therefore, it is important to diagnose AF after an ischemic stroke to provide maximal stroke prevention therapy. Current standard of care dictates an admission electrocardiogram (ECG) and at least 24 h of continuous telemetry monitoring [5]. However, brief asymptomatic paroxysmal atrial fibrillation events may remain undetected by traditional methods of screening. Recent technological advances have made it possible to perform long-term cardiac rhythm monitoring up to months or even years after a stroke. 

## 2. Definition

Paroxysmal atrial fibrillation is not clearly defined in the literature. There is controversy over the duration and morphology of the ECG data in defining an event qualifying for atrial fibrillation. Studies evaluating the incidence of PAF in stroke and TIA patient populations have used different definitions adding confusion about the true incidence. In our paper, we have highlighted the need for a rigorous definition of paroxysmal atrial fibrillation especially in the light of widely used advanced rhythm monitoring devices. 

## 3. Epidemiology

Atrial fibrillation prevalence is associated with age with 0.5% at 50–59 years of age increasing to 8.8% at 80–89 years [2]. PAF comprises from 25% to 60% of these cases [2]. The true incidence of PAF is unclear as most of the prevalence studies used symptomatic events, and prolonged rhythm monitoring was not available at the time on these studies. Moreover, again the variable definitions might have an impact of the incidence reported. 

## 4. Pathophysiology

Atrial arrhythmias have varied pathophysiology ranging from rapidly discharging foci, microreentry, macroreentry, and autonomic modulation of the atria. These processes are usually due to structural abnormality within the atria. This leads to mechanical dysfunction with resultant thrombus formation in the complex, pectinate-rich structure of the left atrial appendage.

## 5. Natural History

Paroxysmal atrial fibrillation is a self-promoting process, and if these events are left untreated, they can progress to persistent atrial fibrillation [3]. The goal of therapy is to maintain sinus rhythm and appropriate anticoagulation [3]. 

## 6. Studies

Multiple studies have been published recently highlighting higher yield of atrial fibrillation detection with longer monitoring and newer devices [6–16]. The yield varied (5%–28%) depending on the choice of monitoring devices, study population, stroke characteristics, interval of monitoring, from stroke onset, duration of cardiac monitoring and most importantly the definition of paroxysmal atrial fibrillation.  Table 1 provides a snap shot of these studies. 

Most of these studies defined paroxysmal atrial fibrillation as events lasting more than 30 seconds. The 30 seconds benchmark used to describe atrial fibrillation events comes from the AHA 2006 guidelines [17]. It is unclear from the manuscript how the authors came up with the 30 seconds benchmark although they do mention that shorter events may be relevant in the right clinical setting [17]. It has been suggested that the duration of atrial fibrillation events with higher specificity can be up to 5 minutes. But then again, this higher specificity comes at the cost of lower sensitivity, and a critical balance between the two needs to be established [18]. 

It is also to be kept in mind that most of these studies are hospital-based identification of stroke/TIA patients with prolonged rhythm monitoring at the discretion of treating physician, and the data was analyzed retrospectively. Therefore, these studies are not free from selection bias. 

It has been shown that these event monitors do have a high false positive rate especially for short events. More refined software with better algorithms to filter out myopotential artifacts can increase specificity, but there is always the risk of losing the sensitivity of event detection [19].

This brings us to the question of what duration of atrial fibrillation events is predictive of a future stroke. No clear association between the duration of events and stroke risk has been established in the literature. It has been shown that even excessive supraventricular ectopic beats not constituting atrial fibrillation increase ischemic stroke and atrial fibrillation detection rates [20]. It is unclear how often these short atrial fibrillation events lead to chronic atrial fibrillation. However, it has also been noted that higher (>5.5 hours) burden of atrial fibrillation events (total duration) leads to a higher stroke risk although this total duration was extrapolated from a long duration of monitoring (>365 days) [21]. Additionally, a recent study showed increased ischemic stroke rates, as well as verified atrial fibrillation rates, in patients that were found to have subclinical atrial tachyarrhythmias of >6 mins [22].

The ongoing study CRYSTAL AF is investigating the value of longer-term monitoring with an implantable loop recorder in patients with cryptogenic stroke to identify the predictive value of these events. Moreover, both CRYSTAL AF and IMPACT trials will help us to identify the best candidates for anticoagulation [23, 24]. IMPACT is also evaluating the therapeutic intervention implied after detection of these events which is the final goal of any diagnostic evaluation in secondary stroke prevention. 

## 7. Predictors 

Multiple predictors of PAF in cryptogenic stroke patients have been proposed by these studies including diabetes, female gender, premature atrial complexes (PACs) on ECG, left atrial dilatation, left ventricular reduced ejection fraction (EF), higher stroke severity assessed by National Institute of Health Stroke Scale (NIHSS), nonlacunar anterior circulation infarcts on neuroimaging, cortical infarcts on neuroimaging, and congestive heart failure [10–12, 14]. Of note, symptoms are not a significant predictor of detecting PAF as most of these events are asymptomatic [25].  Table 2 provides a synopsis of these predictors. 

Most of these predictors make pathophysiological sense because of their ability to modify the atrial rhythms eventually leading to atrial fibrillation. Dilated left atrium and premature atrial complexes stand out as the most important predictors. Transthoracic echocardiogram parameters are useful in selecting the patients for prolonged monitoring in this regard [26]. Premature atrial complexes (PACs) are an important predictor, and a careful analysis of admission ECG should be performed on all stroke patients to identify these PACs for long-term monitoring selection [27].

These various predictors have been consolidated into risk assessment scoring schemes to provide a predictive model for PAF detection [28, 29]. These scores are useful tools, but clinical judgment should always be implied in selecting patients for long-term monitoring. 

## 8. Future Direction

There is a novel concept of atrial fibrillation density which implies temporal clustering of atrial fibrillation events in a short period of time. Essentially, a patient who is monitored in the time window when most of these events are clustering has a higher probability of being detected with monitoring devices as compared to a window period when the events are not clustered. This concept again reaffirms the need to monitor these patients for longer duration [30].

The new subcutaneous implantable cardiac monitors (REVEAL) have shown potential to detect paroxysmal atrial fibrillation with higher patient compliance [31] and longer duration of monitoring. One attractive feature of this device is its MRI compatibility [32]. 

So far, there has been only one randomized clinical trial performed to evaluate the impact of prolonged monitoring on therapeutic decisions. The trial failed to show any benefit of long-term monitoring over routine clinical followup but the sample size of this trial was very small, and the findings need to be validated in a larger clinical study [33].

The iPhone 4S has been shown to potentially act as a monitoring device for detecting PAF and has broad worldwide implications as a method of bringing monitoring to the masses [34].

Multiple genetic polymorphisms have been implicated in pathogenesis of atrial fibrillation such as PITX2, ZFHX3, KCNN3, PRRX1, CAV1, SYNE2, FBP, HCN4, SYNPO2L, and MYOZ1. 

These genes mostly encode for proteins important in the integrity of cardiac myocyte structure and normal physiology [35]. It has been noted that these genetic markers do not increase the detection yield of atrial fibrillation when added to conventional risk factors for atrial fibrillation. Further studies are needed to establish the clinical implication of genetic testing for atrial fibrillation [36].

## 9. Conclusions

Long-term cardiac rhythm monitoring plays an increasingly important role in determining the etiology of stroke/TIA. The widespread use of these monitors will increase the incidence of PAF, raising questions about management of these events. Future studies should focus on the optimal duration of monitoring, predictors of detecting atrial fibrillation, stroke risk pertinent to paroxysmal atrial fibrillation, and the duration of these events. There is also a need to study how often the short atrial fibrillation events lead to chronic atrial fibrillation and optimal treatment strategy, either antiplatelet or anticoagulation, in patients exhibiting brief events.

## Figures and Tables

**Table 1:** Yield of long-term cardiac rhythm monitoring studies.

Study	Patient population	Duration (days)	Sample size	No. diagnosed	Percentage
Barth$\overset{´}{\text{e}}$l$\overset{´}{\text{e}}$my et al. 2003 [6]	Stroke/TIA	4	28	4	14.3
Sposato et al. 2012 [7]	Stroke/TIA	5	155	21	13.5
Stahrenberg et al. 2010 [8]	Stroke/TIA	7	220	28	12.7
Jabaudon et al. 2004 [9]	Stroke/TIA	7	88	5	5.7
Tayal et al. 2008 [10]	Stroke/TIA	21	56	13	23
Miller et al. 2013 [11]	Stroke/TIA	21	156	27	17.3
Bhatt et al. 2011 [12]	Stroke/TIA	21	62	15	24
Elijovich et al. 2009 [13]	Stroke/TIA	30	20	4	20
Gaillard et al. 2010 [14]	Stroke/TIA	30	98	9	9.2
Flint et al. 2012 [15]	Stroke	30	239	29	12.1
Ziegler et al. 2010 [16]	Stroke/TIA	365	163	45	28

Total			1285	200	15.5

**Table 2:** Predictors of atrial fibrillation detection in patients with cryptogenic stroke.

Study	Predictors of atrial fibrillation detection
Tayal et al. 2008 [10]	Diabetes

Miller at al. 2013 [11]	Female gender
	PAC on ECG
	Left atrial dilatation
	Left ventricular EF reduction
	NIHSS

Gaillard et al. 2010 [14]	>100 PACs on 24 hr Holter
	Nonlacunar anterior circulation acute infarcts

Bhatt et al. 2011 [12]	Multiple acute infarcts
	Premature ventricular complexes
